# Non-contiguous finished genome sequence and description of *Bacillus massilioanorexius* sp. nov.

**DOI:** 10.4056/sigs.4087826

**Published:** 2013-07-30

**Authors:** Ajay Kumar Mishra, Anne Pfleiderer, Jean-Christophe Lagier, Catherine Robert, Didier Raoult, Pierre-Edouard Fournier

**Affiliations:** 1 Aix-Marseille Université, URMITE, Faculté de médecine, France

**Keywords:** *Bacillus massilioanorexius*, genome, culturomics, taxonogenomics

## Abstract

*Bacillus massilioanorexius* strain AP8^T^ sp. nov. is the type strain of *B. massilioanorexius* sp. nov., a new species within the genus *Bacillus*. This strain, whose genome is described here, was isolated from the fecal flora of a 21-year-old Caucasian French female suffering from a severe form of anorexia nervosa since the age of 12 years. *B. massilioanorexius* is a Gram-positive aerobic bacillus. Here we describe the features of this organism, together with the complete genome sequence and annotation. The 4,616,135 bp long genome (one chromosome but no plasmid) contains 4,432 protein-coding and 87 RNA genes, including 8 rRNA genes.

## Introduction

*Bacillus massilioanorexius* strain AP8^T^ (= CSUR P201 = DSM 26092) is the type strain of *B. massilioanorexius* sp. nov. This bacterium is a Gram-positive, non-spore-forming, aerobic and motile bacillus that was isolated from the stool of a 21-year-old Caucasian French female suffering from a severe form of anorexia nervosa since the age of 12 years and is part of a “culturomics” study aiming at cultivating all species within human feces individually [1-3]. This bacterium was one of the 11 new bacterial species isolated from this single stool sample [3].

The current classification of *Bacteria* and *Archaea* remains a subject of debate and currently relies on a combination of phenotypic and genotypic characteristics [4]. Genomic data has not yet been routinely incorporated into descriptions. However, as more than 6,000 bacterial genomes have been sequenced including 982 type strains [5,6] and another 15,000 genomic projects are ongoing including 2,120 type strains [5,6], we recently proposed to integrate genomic information in the description of new bacterial species [7-28].

The genus *Bacillus* (Cohn 1872) was created in 1872 [29]. It consists mainly of Gram-positive, motile, spore-forming bacteria classified within 251 species and 3 subspecies with validly published names [30]. Members of the genus *Bacillus* are ubiquitous bacteria isolated from various environments including soil, fresh and sea water and food. In humans, *Bacillus* species may be opportunists in immunocompromised patients [31] or pathogenic, such as *B. anthracis* [32] and *B. cereus*. However, in addition to these two species, various *Bacillus* species may be involved in a variety of aspecific human infections, including cutaneous, ocular, central nervous system or bone infections, pneumonia, endocarditis and bacteremia [33].

Here we present a summary classification and a set of features for *B. massilioanorexius* sp. nov. strain AP8^T^ (= CSUR P201 = DSM 26092), together with the description of the complete genomic sequence and its annotation. These characteristics support the circumscription of the species *B. massilioanorexius*.

## Classification and information

**Figure 1:**
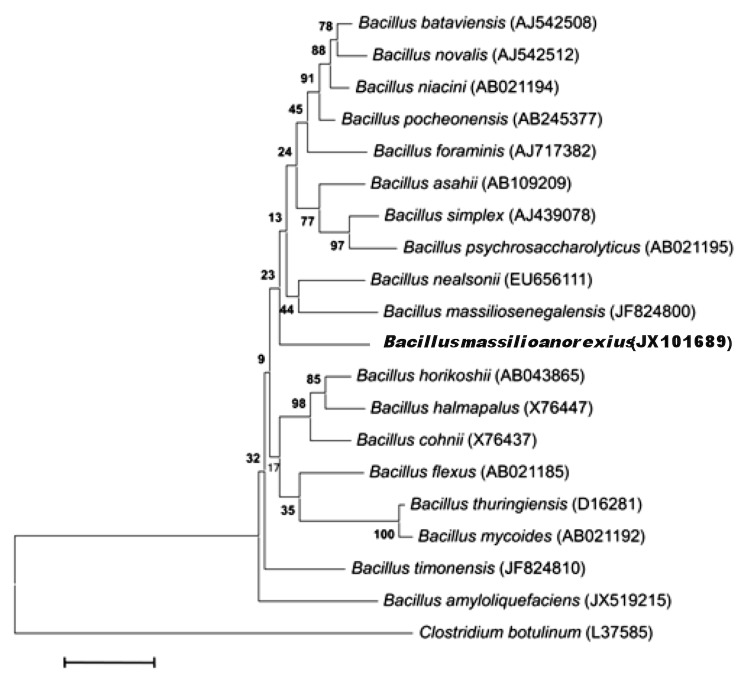
Phylogenetic tree highlighting the position of *Bacillus massilioanorexius* strain AP8^T^ relative to a selection of type strains of validly published species of *Bacillus* genus. GenBank accession numbers are indicated in parentheses. Sequences were aligned using CLUSTALW, and phylogenetic inferences obtained using the maximum-likelihood method within MEGA program. Numbers at the nodes are percentages of bootstrap values obtained by repeating the analysis 500 times to generate a majority consensus tree. *Clostridium botulinum* was used as outgroup. The scale bar represents a 2% nucleotide sequence divergence.

A stool sample was collected from a 21-year-old Caucasian French female suffering from a severe restrictive form of anorexia nervosa since the age of 12 years. She was hospitalized in the nutrition unit of our hospital for recent aggravation of her medical condition. At the time of hospitalization, her weight and height was 27.7 kg, and 1.63 m (BMI: 10.4 kg/m^2^) respectively. The patient gave an informed and signed consent. This study and the assent procedure were approved by the Ethics Committee of the Institut Fédératif de Recherche IFR48, Faculty of Medicine, Marseille, France (agreement 09-022). The fecal specimen was preserved at -80°C after collection. Strain AP8^T^ (Table 1) was isolated in March 2012 by aerobic cultivation on Columbia agar (BioMerieux, Marcy l’Etoile, France) after one month of preincubation of the stool sample with addition of 5ml of sheep rumen in blood bottle culture. This strain exhibited a 97% nucleotide sequence similarity with *B. simplex* [34], the phylogenetically closest validated *Bacillus* species (Figure 1). This value was lower than the 98.7% 16S rRNA gene sequence threshold recommended by Stackebrandt and Ebers to delineate a new species without carrying out DNA-DNA hybridization [35].

**Table 1:** Classification and general features of *Bacillus massilioanorexius* strain AP8^T^

**MIGS ID**	**Property**	**Term**	**Evidence code^a^**
		Domain *Bacteria*	TAS [36]
		Phylum *Firmicutes*	TAS [37-39]
		Class *Bacilli*	TAS [40,41]
	Current classification	Order *Bacillales*	TAS [42,43]
		Family *Bacillaceae*	TAS [42,44]
		Genus *Bacillus*	TAS [29,42,45]
		Species *Bacillus massilioanorexius*	IDA
		Type strain AP8^T^	IDA
	Gram stain	Positive	IDA
	Cell shape	Bacilli	IDA
	Motility	Motile	IDA
	Sporulation	Nonsporulating	IDA
	Temperature range	Mesophile	IDA
	Optimum temperature	37°C	IDA
MIGS-6.3	Salinity	Unknown	IDA
MIGS-22	Oxygen requirement	Aerobic	IDA
	Carbon source	Unknown	NAS
	Energy source	Unknown	NAS
MIGS-6	Habitat	Human gut	IDA
MIGS-15	Biotic relationship	Free living	IDA
MIGS-14	Pathogenicity Biosafety level Isolation	Unknown 2 Human feces	
MIGS-4	Geographic location	France	IDA
MIGS-5	Sample collection time	August 2011	IDA
MIGS-4.1	Latitude Longitude	43.296482 5.36978	IDA IDA
MIGS-4.3	Depth	Surface	IDA
MIGS-4.4	Altitude	0 m above sea level	IDA

Evidence codes - IDA: Inferred from Direct Assay; TAS: Traceable Author Statement (i.e., a direct report exists in the literature); NAS: Non-traceable Author Statement (i.e., not directly observed for the living, isolated sample, but based on a generally accepted property for the species, or anecdotal evidence). These evidence codes are from the Gene Ontology project [46]. If the evidence is IDA, then the property was directly observed for a live isolate by one of the authors or an expert mentioned in the acknowledgements.

**Figure 2:**
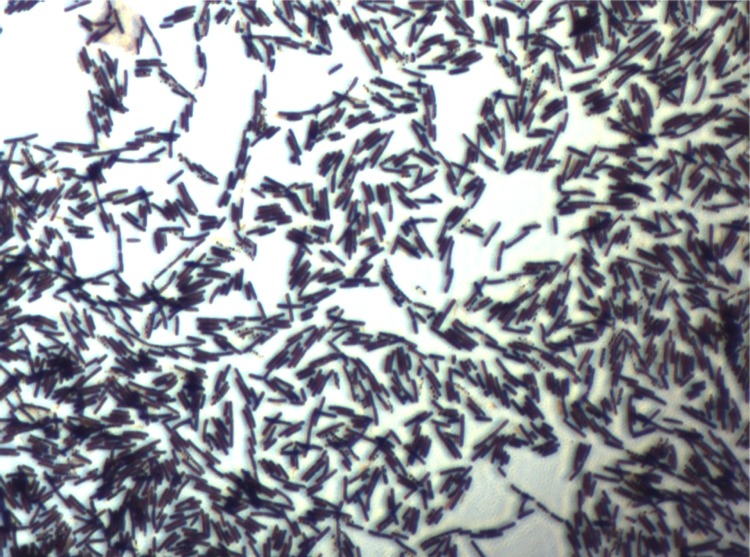
Gram staining of *B. massilioanorexius* strain AP8^T^

**Figure 3:**
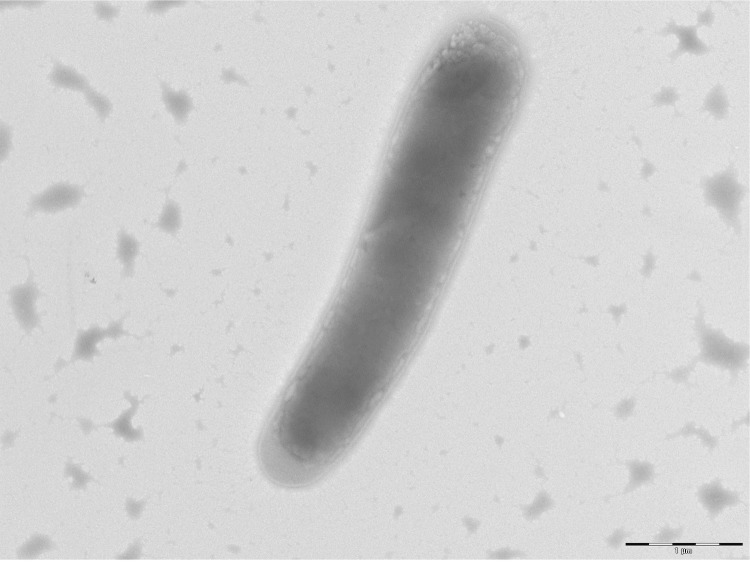
Transmission electron microscopy of *B. massilioanorexius* strain AP8^T^, using a Morgani 268D (Philips) at an operating voltage of 60kV. The scale bar represents 900 nm.

Different growth temperatures (25, 30, 37, 45°C) were tested. Growth was observed between 25 and 45°C, with optimal growth at 37°C after 24 hours of incubation. Colonies were 3 mm in diameter and 0.5 mm in thickness and gray in color with coarse appearance on blood-enriched Columbia agar. Growth of the strain was tested under anaerobic and microaerophilic conditions using GENbag anaer and GENbag microaer systems, respectively (BioMerieux), and under aerobic conditions, with or without 5% CO_2_. Growth was obtained in all the above mentioned conditions except in anaerobic conditions, where weak growth was observed. Gram staining showed Gram-positive rods. The motility test was positive. Cells grown on agar are Gram-positive rods (Figure 2), have a mean diameter of 0.77 µm and a mean length of 2.27 µm in electron microscopy (Figure 3).

Strain AP8^T^ exhibited catalase and oxidase activity. Substrates oxidation and assimilation were examined with an API 50CH strip (BioMerieux) at the optimal growth temperature. Positive reactions were observed for D-glucose, D-fructose, D-saccharose, ribose, mannose, mannitol and D-trehalose and weak reactions were observed for L-rhamnose, esculine, salicine, D-cellobiose and gentiobiose. Using an API 20E strip (BioMerieux, Marcy l’Etoile), positive reactions were observed for tryptophane deaminase, acetoin and gelatinase production. Negative reactions were found for urease and indole production.

*B. massilioanorexius* is susceptible to amoxicillin, rifampicin, ciprofloxacin, gentamicin, doxycycline and vancomycin but resistant to trimethoprim/sulfamethoxazole and metronidazole. When compared with representative species from the genus *Bacillus*, *B. massilioanorexius* strain AP8^T^ exhibited the phenotypic differences detailed in Table 2.

**Table 2:** Differential characteristics of *Bacillus massilioanorexius* strain AP8^T^, *B. timonensis* strain DSM 25372, *B. amyloliquefaciens* strain FZB42, *B. massiliosenegalensis* strain JC6^T^, *B. mycoides* strain DSM 2048 and *B. thuringiensis* strain BMB171

**Properties**	*B. massilioanorexius*	*B. timonensis*	*B. amyloliquefaciens*	*B. massiliosenegalensis*	*B. mycoides*	*B.thuringiensis*
Cell diameter (µm)	0.77	0.66	0.8	0.65	1.1	1.0
Oxygen requirement	aerobic	aerobic	aerobic	aerobic	facultative anaerobic	facultative anaerobic
Pigment production	+	–	–	–	–	–
Gram stain	+	–	+	+	+	+
Salt requirement	–	+	+	+	+	
Motility	+	+	+	+		–
Endospore formation	–	+	+	+	+	+
**Production of**						
Acid phosphatase	na	na	+	w	+	+
Catalase	+	–	+	+	+	+
Oxidase	+	+	+	–	–	+
Nitrate reductase	na	na	+	+	v	+
Urease	–	na	–	-	v	+
β-galactosidase	na	+	v	na	+	–
N-acetyl-glucosamine	na	+	+	+	+	+
**Acid from**						
L-Arabinose	–	+	+	–	+	na
Ribose	+	–	+	–	+	+
Mannose	+	–	+	–	+	+
Mannitol	+	–	+	–	+	+
Sucrose	–	–	+	–	+	v
D-glucose	+	–	+	+	+	+
D-fructose	+	–	+	–	+	+
D-maltose	–	–	+	+	+	+
D-lactose	–	+	+	–	+	+
**Hydrolysis of**						
Gelatin	+	–	+	–	+	+
Starch	na	na	+	na	+	+
**G+C content (mol%)**	34.10	37.30	46.48	37.6	35.21	35.18
**Habitat**	human gut	human gut	Soil	human gut	soil	soil

na = data not available; w = weak, v = variable reaction

**Figure 4:**
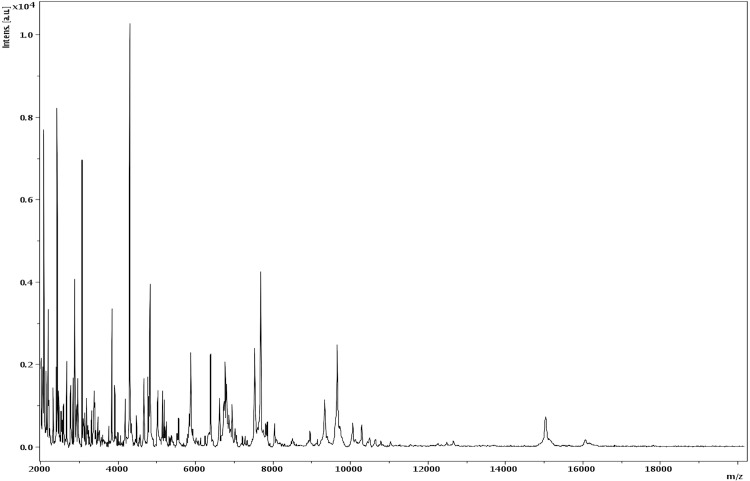
Reference mass spectrum from *B. massilioanorexius* strain AP8^T^. Spectra from 12 individual colonies were compared and a reference spectrum was generated.

**Figure 5:**
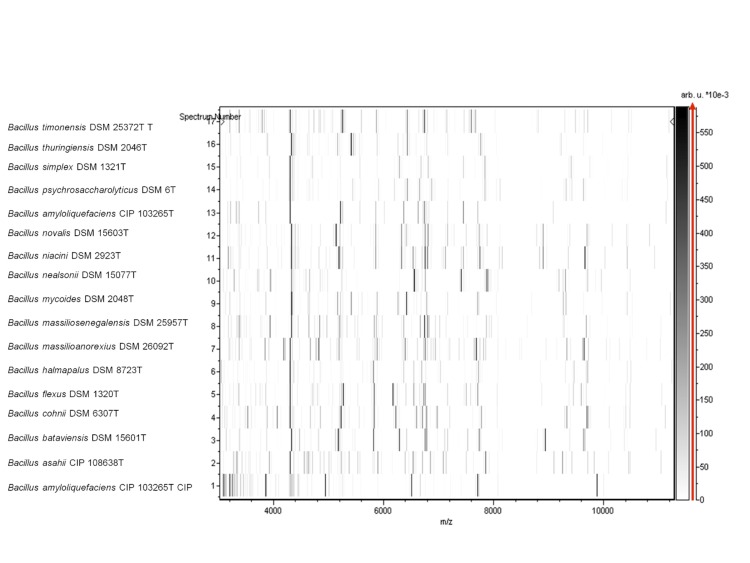
Gel view comparing *B. massilioanorexius* sp. nov strain AP8^T^ and other *Bacillus* species. The gel view displays the raw spectra of loaded spectrum files arranged in a pseudo-gel like look. The x-axis records the m/z value. The left y-axis displays the running spectrum number originating from subsequent spectra loading. The peak intensity is expressed by a Gray scale scheme code. The color bar and the right y-axis indicate the relation between the color a peak is displayed with and the peak intensity in arbitrary units. Displayed species are indicated on the left.

Matrix-assisted laser-desorption/ionization time-of-flight (MALDI-TOF) MS protein analysis was carried out as previously described [47] using a Microflex spectrometer (Brüker Daltonics, Leipzig, Germany). Twelve individual colonies were deposited on a MTP 384 MALDI-TOF target plate (Brüker). The twelve AP8^T^ spectra were imported into the MALDI BioTyper software (version 2.0, Brüker) and analyzed by standard pattern matching (with default parameter settings) against the main spectra of 3,769 bacteria, including 129 spectra from 98 validly named *Bacillus* species, used as reference data in the BioTyper database. A score enabled the presumptive identification and discrimination of the tested species from those in a database: a score > 2 with a validated species enabled the identification at the species level; and a score < 1.7 did not enable any identification. For strain AP8^T^, no significant score was obtained, suggesting that our isolate was not a member of any known species (Figures 4 and 5).

## Genome sequencing information

### Genome project history

The organism was selected for sequencing on the basis of its phylogenetic position and 16S rRNA similarity to other members of the *Bacillus* genus, and is part of a “culturomics” study of the human digestive flora aiming at isolating all bacterial species within human feces. It was the twenty-seventh genome of a *Bacillus* species and the first genome of *Bacillus massilioanorexius* sp. nov. A summary of the project information is shown in Table 3. The Genbank accession number is CAPG00000000 and consists of 120 contigs. Table 3 shows the project information and its association with MIGS version 2.0 compliance [48].

**Table 3:** Project information

**MIGS ID**	**Property**	**Term**
MIGS-31	Finishing quality	High-quality draft
MIGS-28	Libraries used	One 454 paired end 3-kb library
MIGS-29	Sequencing platforms	454 GS FLX Titanium
MIGS-31.2	Fold coverage	31.34 ×
MIGS-30	Assemblers	Newbler version 2.5.3
MIGS-32	Gene calling method	Prodigal
	Genbank ID	CAPG00000000
	Genbank Date of Release	November 28, 2012
	Gold ID	Gi20708
MIGS-13	Project relevance	Study of the human gut microbiome

### Growth conditions and DNA isolation

Strain AP8^T^ was grown aerobically in Columbia broth (BioMerieux, Marcy l’Etoile, France). Extraction of chromosomal DNA was performed by using 50 mL of 48-72 h culture of *B. massilioanorexius*, centrifuged at 4^o^C and 2000 × g for 20 min. Resuspension of cell pellets was done in 1 mL Tris/EDTA/NaCl [10mM Tris/HCl (pH7.0), 10 mM EDTA (pH8.0), and 300 mM NaCl] and re-centrifugation was done under the same conditions. The pellets were resuspended in 200µL TE/lysozyme [25 mM Tris/HCl (pH8.0), 10 mM EDTA (pH8.0), 10 mM NaCl, and 10 mg lysozyme/mL]. The sample was incubated at 37^o^C for 30 min and then 30 µL of 30% (w/v) sodium N- lauroyl-sarcosine (Sarcosyl) was added to it, incubated for 20 min at 65^o^C, followed by incubation for 5 min at 4^o^C. Purification of DNA with phenol/chloroform/isoamylalcohol (25:24:1) was followed by precipitation with ethanol. DNA concentration was 64.3 ng/µl as determined by Genios Tecan fluorometer, using the Quant-it Picogreen kit (Invitrogen).

### Genome sequencing and assembly

A 3kb paired-end sequencing strategy (Roche, Meylan, France) was used. Five µg of DNA were mechanically fragmented on the Covaris device (KBioScience-LGC Genomics, Middlesex, UK) through miniTUBE-Red with an enrichment size at 3-4kb. The DNA fragmentation was visualized through the Agilent 2100 BioAnalyzer on a DNA labchip 7500 with an optimal size of 2.95 kb. The library was constructed according to the 454 GS FLX Titanium paired end protocol. Circularization and nebulization were performed which generated a pattern of 553 bp optimal size. PCR amplification was performed for 17 cycles followed by double size selection. The single stranded paired-end library was quantified using Quant-it Ribogreen kit (Invitrogen) with Genios Tecan fluorometer that yielded concentration of 556 pg/µL. The library concentration equivalence was calculated as 1.82E+09 molecules/µL. The library was stored at -20°C until further use.

The shotgun library was clonally amplified with 5cpb in 4 emPCR reactions and the 3kb paired-end library was amplified with lower cpb in 4 emPCR reactions at 1cpb and 2 emPCR at 0.5 cpb with the GS Titanium SV emPCR Kit (Lib-L) v2 (Roche). The yield of the shotgun emPCR reactions was 16.9 and 5.62% respectively for the two kinds of paired-end emPCR reactions according to the quality expected (range of 5 to 20%) from the Roche procedure. Two libraries were loaded on the GS Titanium PicoTiterPlates (PTP Kit 70x75, Roche) and pyrosequenced with the GS Titanium Sequencing Kit XLR70 and the GS FLX Titanium sequencer (Roche). The run was performed overnight and analyzed on the cluster through the gsRunBrowser and Newbler assembler (Roche). A total of 410,883 passed filter wells were obtained and generated 144.49 Mb with a length average of 344 bp. The passed filter sequences were assembled Using Newbler with 90% identity and 40 bp as overlap. The final assembly identified 20 scaffolds and 120 contigs and generated a genome size of 4.61Mb which corresponds to a coverage of 31.34 × genome equivalent.

### Genome annotation

Open Reading Frames (ORFs) were predicted using Prodigal [49] with default parameters but the predicted ORFs were excluded if they were spanning a sequencing gap region. The predicted bacterial protein sequences were searched against the GenBank database [50] and the Clusters of Orthologous Groups (COG) databases using BLASTP. The tRNAScanSE tool [51] was used to find tRNA genes, whereas ribosomal RNAs were found by using RNAmmer [52] and BLASTn against the GenBank database. Lipoprotein signal peptides and the number of transmembrane helices were predicted using SignalP [53] and TMHMM [54] respectively. ORFans were identified if their BLASTP *E*-value was lower than 1e^-03^ for alignment length greater than 80 amino acids. If alignment lengths were smaller than 80 amino acids, we used an *E*-value of 1e^-05^. Such parameter thresholds have already been used in previous works to define ORFans. Ortholog sets composed of one gene from each of six genomes (*B. massilioanorexius* strain AP8^T^, *B. timonensis* strain DSM 25372 (GenBank accession number CAET00000000), *B. amyloliquefaciens* strain FZB42 (GenBank accession number NC_009725), *B. massiliosenegalensis* strain JC6^T^ (GenBank accession number CAHJ00000000), *B. mycoides* strain DSM 2048 (GenBank accession number CM000742) and *B. thuringiensis* strain BMB171 (GenBank accession number CP001903),) were identified using the Proteinortho software (version 1.4) [55] using a 30% protein identity and 1e^-05^ *E*-value. The average percentage of nucleotide sequence identity between corresponding orthologous sets were determined using the Needleman-Wunsch algorithm global alignment technique.

Artemis [56] was used for data management and DNA Plotter [57] was used for visualization of genomic features. Mauve alignment tool was used for multiple genomic sequence alignment and visualization [58].

## Genome properties

**Figure 6:**
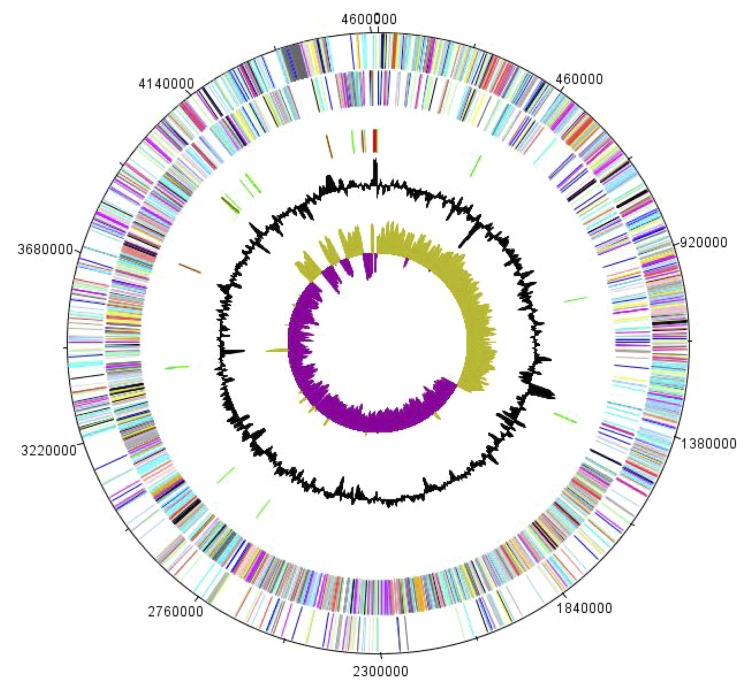
Graphical circular map of the chromosome. From the outside in, the outer two circles show open reading frames oriented in the forward and reverse directions (colored by COG categories), respectively. The third circle shows the rRNA gene operon (red) and tRNA genes (green). The fourth circle shows the G+C% content plot. The inner-most circle shows GC skew, purple and olive indicating negative and positive values, respectively.

The genome of *B. massiliensis* strain AP8^T^ is 4,616,135 bp long (1 chromosome, but no plasmid) with a 34.10% G + C content (Figure 6 and Table 4). Of the 4,519 predicted genes, 4,432 were protein-coding genes, and 87 were RNAs. Eight rRNA genes (one 16S rRNA, one 23S rRNA and six 5S rRNA) and 79 predicted tRNA genes were identified in the genome. A total of 3,290 genes (72.80%) were assigned a putative function. Three hundred fifty-four genes were identified as ORFans (7.98%). The remaining genes were annotated as hypothetical proteins. The properties and the statistics of the genome are summarized in Table 4 and Table 5. The distribution of genes into COGs functional categories is presented in Table 5.

**Table 4:** Nucleotide content and gene count levels of the genome

Attribute	Value	% of total^a^
Genome size (bp)	4,616,135	
DNA coding region (bp)	3,750,534	81.24
DNA G+C content (bp)	1,574,102	34.10
Number of replicons	1	
Extrachromosomal elements	0	
Total genes	4,519	100
RNA genes	87	1.92
rRNA operons	1	
Protein-coding genes	4,432	98.07
Genes with function prediction	3,524	77.98
Genes assigned to COGs	3,290	72.80
Protein coding genes assigned Pfam domains	3,807	84.24
Genes with peptide signals	270	5.97
Genes with transmembrane helices	1,241	27.46
CRISPR repeats	2	

^a^ The total is based on either the size of the genome in base pairs or the total number of protein coding genes in the annotated genome

**Table 5:** Number of genes associated with the 25 general COG functional categories

**Code**	**Value**	**% of total**^a^	**Description**
J	171	3.86	Translation
A	0	0	RNA processing and modification
K	335	7.56	Transcription
L	200	4.51	Replication, recombination and repair
B	1	0.02	Chromatin structure and dynamics
D	37	0.83	Cell cycle control, mitosis and meiosis
Y	0	0	Nuclear structure
V	76	1.71	Defense mechanisms
T	212	4.78	Signal transduction mechanisms
M	147	3.32	Cell wall/membrane biogenesis
N	70	1.58	Cell motility
Z	0	0	Cytoskeleton
W	0	0	Extracellular structures
U	48	1.08	Intracellular trafficking and secretion
O	121	2.73	Posttranslational modification, protein turnover, chaperones
C	245	5.53	Energy production and conversion
G	221	4.99	Carbohydrate transport and metabolism
E	405	9.14	Amino acid transport and metabolism
F	98	2.21	Nucleotide transport and metabolism
H	135	3.05	Coenzyme transport and metabolism
I	136	3.07	Lipid transport and metabolism
P	258	5.82	Inorganic ion transport and metabolism
Q	81	1.83	Secondary metabolites biosynthesis, transport and catabolism
R	527	11.89	General function prediction only
S	349	7.87	Function unknown
-	1,142	25.77	Not in COGs

^a^ The total is based on the total number of protein coding genes in the annotated genome.

## Comparison with other *Bacillus* species genomes

**Figure 7:**
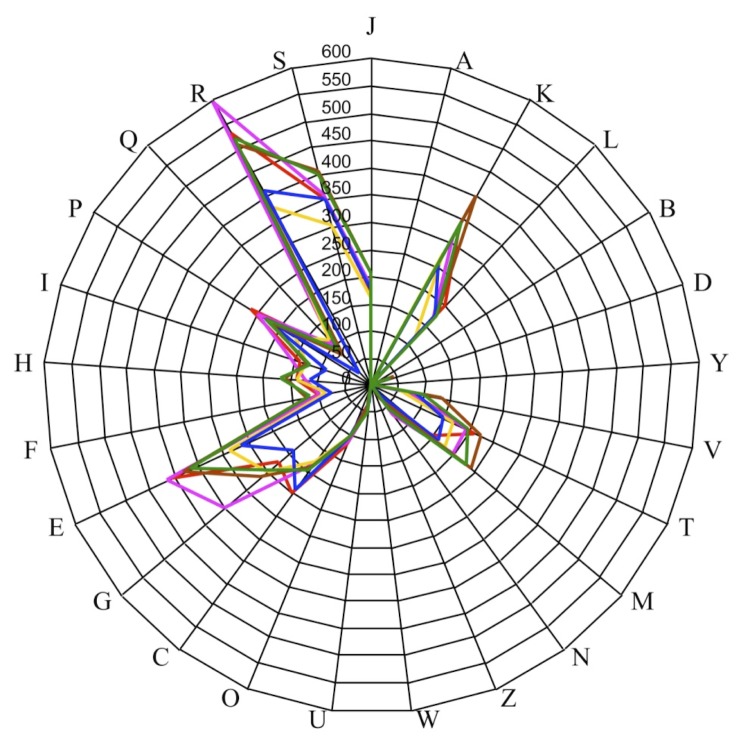
Distribution of functional classes of predicted genes in *B. massilioanorexius* (red), *B. massiliosenegalensis* (blue), *B. timonensis* (pink), *B. amyloliquefaciens* (yellow), *B. mycoides* (brown) and *B. thuringiensis* (green) chromosomes according to the clusters of orthologous groups of proteins.

Here, we compared the genome of *B. massilioanorexius* strain AP8^T^, *B. timonensis* strain DSM 25372, *B. amyloliquefaciens* strain FZB42, *B. massiliosenegalensis* strain JC6^T^, *B. mycoides* strain DSM 2048 and *B. thuringiensis* strain BMB171. The draft genome of *B. massilioanorexius* is larger in size than that of *B. amyloliquefaciens* (4.6 vs 3.9 Mb, respectively), similar in size than that of *B. timonensis* (4.6 Mb) and smaller in size than those of *B. massiliosenegalensis*, *B. mycoides* and *B. thuringiensis* (4.9, 5.5 and 5.6 Mb, respectively). The G+C content of *B. massilioanorexius* is lower than those of *B. massiliosenegalensis*, *B. timonensis*, *B. amyloliquefaciens*, *B. mycoides* and *B. thuringiensis* (34.10, 37.60, 37.30, 46.48, 35.21 and 35.18%, respectively). The gene content of *B. massilioanorexius* is larger than that of *B. amyloliquefaciens* (4,519 and 3,814, respectively) and fewer than those of *B. massiliosenegalensis*, *B. timonensis*, *B. mycoides* and *B. thuringiensis* (4,997, 4,684, 5,747 and 5,495, respectively). The ratio of genes per MB of *B. massilioanorexius* is greater than that of *B. amyloliquefaciens* (982 and 978, respectively), comparable to that of *B. thuringiensis* (982) and smaller to those of *B. massiliosenegalensis*, *B. timonensis* and *B. mycoides* (1,019, 1,018 and 1,044, respectively). However, the distribution of genes into COG categories was not entirely similar in all the three compared genomes (Figure 7). The nucleotide sequence identity ranged from 66.09 to 83.69% among *Bacillus* species, and from 66.09 to 70.10% between *B. massilioanorexius* and other *Bacillus* species, thus confirming its new species status. Table 6 summarizes the numbers of orthologous genes and the average percentage of nucleotide sequence identity between the different genomes studied.

**Table 6:** Orthologous gene comparison and average nucleotide identity *Bacillus* species *B. massilioanorexius*^1^ with *B. massiliosenegalensis*^2^; *B. timonensis*^3^, *B. thuringiensis*^4^; *B. mycoides*^5^; *B. amyloliquefaciens*^6†^.

	*B. massilioanorexius*	*B. massiliosenegalensis*	*B. timonensis*	*B. huringiensis*	*B. mycoides*	*B. amyloliquefaciens*
*B. massilioanorexius*	**4,432**	1,897	1,864	1,887	1,794	1,709
*B. massiliosenegalensis*	70.10	**4,895**	1,965	1,863	1,765	1,755
*B. timonensis*	69.84	70.33	**4,610**	1,864	1,762	1,742
*B. thuringiensis*	69.35	68.88	69.31	**6,243**	2,210	1,832
*B. mycoides*	69.41	69.11	69.41	83.69	**5,885**	1,719
*B. amyloliquefaciens*	66.09	67.02	67.12	66.35	66.57	**3,823**

^†^Upper right, numbers of orthologous genes; lower left, mean nucleotide identities of orthologous genes. Bold numbers indicate the numbers of genes or each genome. ^1^Genbank accession number CAPG00000000, ^2^CAHJ00000000, ^3^CAET00000000, ^4^CP001903, ^5^CM000742, ^6^NC_009725

## Conclusion

On the basis of phenotypic, phylogenetic and genomic analyses, we formally propose the creation of *Bacillus massilioanorexius* sp. nov. that contains the strain AP8^T^. The strain has been found in France.

### Description of *Bacillus massilioanorexius* sp. nov.

*Bacillus massilioanorexius* (ma.si.li.o.a.no.rex’i.us. L. masc. adj. massilioanorexius, combination of Massilia, the Latin name of Marseille, France, where the type strain was isolated, and anorexia, the disease presented by the patient from whom the strain was cultivated).

Colonies were 3 mm in diameter and 0.5 mm in thickness, gray in color with a coarse appearance on blood-enriched Columbia agar. Cells are rod-shaped with a mean diameter of 0.77 µm. Optimal growth occurs aerobically, weak growth was observed under anaerobic conditions. Growth occurs between 25 and 45°C, with optimal growth observed at 37°C. Cells stain Gram-positive, are non-endospore forming and are motile. Cells are Gram-positive, catalase-positive, oxidase-positive. D-glucose, D-fructose, D-saccharose, D-trehalose, ribose, mannitol, mannose were used as carbon source. Positive reactions were observed for tryptophane deaminase, acetoin and gelatinase production. Weak reactions were obtained for L-rhamnose, esculine, salicine, D-cellobiose and gentiobiose. Cells are susceptible to amoxicillin, rifampicin, ciprofloxacin, gentamicin, doxycycline and vancomycin but resistant to trimethoprim/sulfamethoxazole and metronidazole.

The G+C content of the genome is 34.10%. The 16S rRNA and genome sequences are deposited in GenBank under accession numbers JX101689 and CAPG00000000, respectively. The type strain AP8^T^ (= CSUR P201 = DSM 26092) was isolated from the fecal flora of a female suffering from anorexia nervosa in Marseille, France.

## References

[1] Lagier JC Armougom F Million M Hugon P Pagnier I Robert C Bittar F Fournous G Gimenez G Maraninchi M Microbial culturomics: paradigm shift in the human gut microbiome study. Clin Microbiol Infect 2012; 18:1185-1193 23033984 10.1111/1469-0691.12023

[2] Dubourg G Lagier JC Armougom F Robert C Hamad I Brouqui P The gut microbiota of a patient with resistant tuberculosis is more comprehensively studied by culturomics than by metagenomics. Eur J Clin Microbiol Infect Dis 2013; 32:637-645 10.1007/s10096-012-1787-3 23291779

[3] Pfleiderer A Lagier JC Armougom F Robert C Vialettes B Raoult D Culturomics identified 11 new bacterial species from a single anorexia nervosa stool sample. [Epub ahead of print]. Eur J Clin Microbiol Infect Dis 2013 10.1007/s10096-013-1900-2 23728738

[4] Tindall BJ Rossello-Mora R Busse HJ Ludwig W Kampfer P Notes on the characterization of prokaryote strains for taxonomic purposes. Int J Syst Evol Microbiol 2010; 60:249-266 10.1099/ijs.0.016949-0 19700448

[5] Genome Online Database http://www.genomesonline.org/cgi-bin/GOLD/index.cgi

[6] The Microbial Earth Project. http://www.microbial-earthorg

[7] Kokcha S Mishra AK Lagier JC Million M Leroy Q Raoult D Fournier PE Non-contiguous finished genome sequence and description of *Bacillus timonensis* sp. nov. Stand Genomic Sci 2012; 6:346-355 10.4056/sigs.2776064 23408487 PMC3558959

[8] Lagier JC El Karkouri K Nguyen TT Armougom F Raoult D Fournier PE Non-contiguous finished genome sequence and description of *Anaerococcus senegalensis* sp. nov. Stand Genomic Sci 2012; 6:116-125 10.4056/sigs.2415480 22675604 PMC3359877

[9] Mishra AK Gimenez G Lagier JC Robert C Raoult D Fournier PE Non-contiguous finished genome sequence and description of *Alistipes senegalensis* sp. nov. Stand Genomic Sci 2012; 6:304-314 10.4056/sigs.2625821 PMC3569391 23407294

[10] Lagier JC Armougom F Mishra AK Ngyuen TT Raoult D Fournier PE Non-contiguous finished genome sequence and description of *Alistipes timonensis* sp. nov. Stand Genomic Sci 2012; 6:315-324 23408657 10.4056/sigs.2685971 PMC3558960

[11] Mishra AK Lagier JC Robert C Raoult D Fournier PE Non-contiguous finished genome sequence and description of *Clostridium senegalense* sp. nov. Stand Genomic Sci 2012; 6:386-395 23408737 10.4056/sigs.2766062 PMC3558962

[12] Mishra AK Lagier JC Robert C Raoult D Fournier PE Non-contiguous finished genome sequence and description of *Peptoniphilus timonensis* sp. nov. Stand Genomic Sci 2012; 7:1-11 10.4056/sigs.2956294 23449949 PMC3570796

[13] Mishra AK Lagier JC Rivet R Raoult D Fournier PE Non-contiguous finished genome sequence and description of *Paenibacillus senegalensis* sp. nov. Stand Genomic Sci 2012; 7:70-81 23459006 10.4056/sigs.3056450 PMC3577113

[14] Lagier JC Gimenez G Robert C Raoult D Fournier PE Non-contiguous finished genome sequence and description of *Herbaspirillum massiliense* sp. nov. Stand Genomic Sci 2012; 7:200-209 23407294 10.4056/sigs.3086474 PMC3569391

[15] Roux V El Karkouri K Lagier JC Robert C Raoult D Non-contiguous finished genome sequence and description of *Kurthia massiliensis* sp. nov. Stand Genomic Sci 2012; 7:221-232 10.4056/sigs.3206554 23407462 PMC3569394

[16] Kokcha S Ramasamy D Lagier JC Robert C Raoult D Fournier PE Non-contiguous finished genome sequence and description of *Brevibacterium senegalense* sp. nov. Stand Genomic Sci 2012; 7:233-245 10.4056/sigs.3256677 23408786 PMC3569389

[17] Ramasamy D Kokcha S Lagier JC N’Guyen TT Raoult D Fournier PE Non-contiguous finished genome sequence and description of *Aeromicrobium massilense* sp. nov. Stand Genomic Sci 2012; 7:246-257 10.4056/sigs.3306717 23408786 PMC3569389

[18] Lagier JC Ramasamy D Rivet R Raoult D Fournier PE Non-contiguous finished genome sequence and description of *Cellulomonas massiliensis* sp. nov. Stand Genomic Sci 2012; 7:258-270 10.4056/sigs.3316719 23408774 PMC3569388

[19] Lagier JC El Karkouri K Rivet R Couderc C Raoult D Fournier PE Non-contiguous finished genome sequence and description of *Senegalemassilia anaerobia* sp. nov. Stand Genomic Sci 2013; 7:343-356 10.4056/sigs.3246665 24019984 PMC3764928

[20] Mishra AK Hugon P Lagier JC Nguyen TT Robert C Couderc C Raoult D Fournier PE Non-contiguous finished genome sequence and description of *Peptoniphilus obesi* sp. nov. Stand Genomic Sci 2013; 7:357-369 10.4056/sigs.32766871 24019985 PMC3764929

[21] Mishra AK Lagier JC Nguyen TT Raoult D Fournier PE Non-contiguous finished genome sequence and description of *Peptoniphilus senegalensis* sp. nov. Stand Genomic Sci 2013; 7:370-381 10.4056/sigs.3366764 24019986 PMC3764932

[22] Lagier JC El Karkouri K Mishra AK Robert C Raoult D Fournier PE Non-contiguous finished genome sequence and description of *Enterobacter massiliensis* sp. nov. Stand Genomic Sci 2013; 7:399-412 10.4056/sigs.3396830 24019988 PMC3764934

[23] Hugon P Ramasamy D Lagier JC Rivet R Couderc C Raoult D Fournier PE Non-contiguous finished genome sequence and description of *Alistipes obesi* sp. nov. Stand Genomic Sci 2013; 7:427-439 10.4056/sigs.3336746 24019990 PMC3764931

[24] Mishra AK Hugon P Robert C Couderc C Raoult D Fournier PE Non-contiguous finished genome sequence and description of *Peptoniphilus grossensis* sp. nov. Stand Genomic Sci 2012; 7:320-330 23408485 10.4056/sigs.3076460 PMC3569384

[25] Mishra AK Hugon P Lagier JC Nguyen TT Couderc C Raoult D Fournier PE Non contiguous-finished genome sequence and description of *Enorma massiliensis* gen. nov., sp. nov., a new member of the Family *Coriobacteriaceae.* Stand Genomic Sci 2013; 8:290-305 10.4056/sigs.3426906 23991260 PMC3746427

[26] Ramasamy D Lagier JC Gorlas A Raoult D Fournier PE Non contiguous-finished genome sequence and description of Bacillus massiliosenegalensis sp. nov. Stand Genomic Sci 2013; 8:336-351 10.4056/sigs.3567059 23991258 PMC3746431

[27] Ramasamy D Lagier JC Nguyen TT Raoult D Fournier PE Non contiguous-finished genome sequence and description of of Dielma fastidiosa gen. nov., sp. nov., a new member of the Family Erysipelotrichaceae. Stand Genomic Sci 2013; 8:336-351 10.4056/sigs.3567059 23991263 PMC3746426

[28] Mishra AK Lagier JC Robert C Raoult D Fournier PE Genome sequence and description of *Timonella senegalensis* gen. nov., sp. nov., a new member of the suborder *Micrococcinae.* Stand Genomic Sci 2013; 8:318-335 10.4056/sigs.3476977 23991262 PMC3746429

[29] Cohn F Untersuchungen über Bakterien. Beitrage zur Biologie der Pflanzen Heft 1872; 1:127-224

[30] Mathews WC, Caperna J, Toerner JG, Barber RE, Morgenstern H. Neutropenia is a risk factor for gram-negative bacillus bacteremia in human immunodeficiency virus-infected patients: results of a nested case-control study. *Am J Epidemiol* 1998; **48**:1175-1183.25. 10.1093/oxfordjournals.aje.a009606 9867263

[31] Logan NA *Bacillus* species of medical and veterinary importance. J Med Microbiol 1988; 25:157-165 10.1099/00222615-25-3-157 3279213

[32] Jernigan JA Stephens DS Ashford DA Omenaca C Topiel MS Galbraith M Tapper M Fisk TL Zaki S Popovic T Bioterrorism-related inhalational anthrax: the first 10 cases reported in the United States. Emerg Infect Dis 2001; 7:933-944 10.3201/eid0706.010604 11747719 PMC2631903

[33] Bottone EJ *Bacillus cereus*, a volatile human pathogen. Clin Microbiol Rev 2010; 23:382-398 10.1128/CMR.00073-09 20375358 PMC2863360

[34] Purswani J Pozo C Rodriguez-Diaz M Gonzalez-Lopez J Selection and identification of bacterial strains with methyl-tert-butyl ether, ethyl-tert-butyl ether, and tert-amyl methyl ether degrading capacities. Environ Toxicol Chem 2008; 27:2296-2303 10.1897/08-096.1 18522454

[35] Stackebrandt E Ebers J Taxonomic parameters revisited: tarnished gold standards. Microbiol Today 2006; 33:152-155

[36] Woese CR Kandler O Wheelis ML Towards a natural system of organisms: proposal for the domains *Archae, Bacteria*, and *Eukarya.* Proc Natl Acad Sci USA 1990; 87:4576-4579 10.1073/pnas.87.12.4576 2112744 PMC54159

[37] Gibbons NE Murray RGE Proposals concerning the Higher Taxa of the Bacteria. Int J Syst Bacteriol 1978; 28:1-6 10.1099/00207713-28-1-1

[38] Garrity GM, Holt JG. The Road Map to the Manual. In: Garrity GM, Boone DR, Castenholz RW (eds), *Bergey's Manual of Systematic Bacteriolog*y, Second Edition, Volume 1, Springer, New York, 2001, p. 119-169.

[39] Murray RGE. The Higher Taxa, or, a Place for Everything...? In: Holt JG (ed), *Bergey's Manual of Systematic Bacteriology*, First Edition, Volume 1, The Williams and Wilkins Co., Baltimore, 1984, p. 31-34.

[40] List Editor List of new names and new combinations previously effectively, but not validly, published. List no. 132. Int J Syst Evol Microbiol 2010; 60:469-472 10.1099/ijs.0.022855-0 20458120

[41] Ludwig W, Schleifer KH, Whitman WB. Class I. *Bacilli* class nov. In: De Vos P, Garrity G, Jones D, Krieg NR, Ludwig W, Rainey FA, Schleifer KH, Whitman WB (eds), *Bergey's Manual of Systematic Bacteriology*, Second Edition, Volume 3, Springer-Verlag, New York, 2009, p. 19-20.

[42] Skerman VBD Sneath PHA Approved list of bacterial names. Int J Syst Bact 1980; 30:225-420 10.1099/00207713-30-1-225

[43] Prévot AR. Dictionnaire des bactéries pathogens. *In*: Hauduroy P, Ehringer G, Guillot G, Magrou J, Prevot AR, Rosset, Urbain A (*eds*). Paris, Masson, 1953, p.1-692.

[44] Fischer A Untersuchungen über bakterien. Jahrbücher für Wissenschaftliche Botanik 1895; 27:1-163

[45] Gibson T, Gordon RE. Genus I. *Bacillus* Cohn 1872, 174; Nom. gen. cons. Nomencl. Comm. Intern. Soc. Microbiol. 1937, 28; Opin. A. Jud. Comm. 1955, 39. In: Buchanan RE, Gibbons NE (eds), *Bergey's Manual of Determinative Bacteriology*, Eighth Edition, The Williams and Wilkins Co., Baltimore, 1974, p. 529-550.

[46] Ashburner M Ball CA Blake JA Botstein D Butler H Cherry JM Davis AP Dolinski K Dwight SS Eppig JT Gene ontology: tool for the unification of biology. The Gene Ontology Consortium. Nat Genet 2000; 25:25-29 10.1038/75556 10802651 PMC3037419

[47] Seng P Drancourt M Gouriet F La Scola B Fournier PE Rolain JM Raoult D Ongoing revolution in bacteriology: routine identification of bacteria by matrix-assisted laser desorption ionization time-of-flight mass spectrometry. Clin Infect Dis 2009; 49:543-551 10.1086/600885 19583519

[48] Field D Garrity G Gray T Morrison N Selengut J Sterk P Tatusova T Thomson N Allen MJ Angiuoli SV The minimum information about a genome sequence (MIGS) specification. Nat Biotechnol 2008; 26:541-547 10.1038/nbt1360 18464787 PMC2409278

[49] Prodigal. http://prodigal.ornl.gov

[50] GenBank database. http://www.ncbi.nlm.nih.gov/genbank

[51] Lowe TM Eddy SR tRNAscan-SE: a program for improved detection of transfer RNA genes in genomic sequence. Nucleic Acids Res 1997; 25:955-964 9023104 10.1093/nar/25.5.955 PMC146525

[52] Lagesen K Hallin P Rodland EA Staerfeldt HH Rognes T Ussery DW RNAmmer: consistent and rapid annotation of ribosomal RNA genes. Nucleic Acids Res 2007; 35:3100-3108 10.1093/nar/gkm160 17452365 PMC1888812

[53] Bendtsen JD Nielsen H von Heijne G Brunak S Improved prediction of signal peptides: SignalP 3.0. J Mol Biol 2004; 340:783-795 10.1016/j.jmb.2004.05.028 15223320

[54] Krogh A Larsson B von Heijne G Sonnhammer EL Predicting transmembrane protein topology with a hidden Markov model: application to complete genomes. J Mol Biol 2001; 305:567-580 10.1006/jmbi.2000.4315 11152613

[55] Lechner M Findeib S Steiner L Marz M Stadler PF Prohaska SJ Proteinortho: Detection of (Co-)orthologs in large-scale analysis. BMC Bioinformatics 2011; 12:124 10.1186/1471-2105-12-124 21526987 PMC3114741

[56] Rutherford K Parkhill J Crook J Horsnell T Rice P Rajandream MA Barrell B Artemis: sequence visualization and annotation. Bioinformatics 2000; 16:944-945 10.1093/bioinformatics/16.10.944 11120685

[57] Carver T Thomson N Bleasby A Berriman M Parkhill J DNAPlotter: circular and linear interactive genome visualization. Bioinformatics 2009; 25:119-120 10.1093/bioinformatics/btn578 18990721 PMC2612626

[58] Darling AC Mau B Blattner FR Perna NT Mauve: multiple alignment of conserved genomic sequence with rearrangements. Genome Res 2004; 14:1394-1403 10.1101/gr.2289704 15231754 PMC442156

